# Electrical Impedance Tomography Monitoring During Extubation in Critically Ill Children

**DOI:** 10.3390/children13020190

**Published:** 2026-01-29

**Authors:** Waratchaya Kit-Anan, Jarin Vaewpanich, Nattachai Anantasit

**Affiliations:** Division of Pediatric Critical Care Medicine, Department of Pediatrics, Ramathibodi Hospital, Mahidol University, Bangkok 10400, Thailand; waratchaya.sur@cpird.in.th (W.K.-A.); jarin.vae@mahidol.ac.th (J.V.)

**Keywords:** electrical impedance tomography, extubation, critical care, technology dependence

## Abstract

**Highlights:**

**What are the main findings?**

• Significant changes observed in key EIT parameters, including ΔEELI, tidal impedance, and global inhomogeneity, were consistently observed across the pre- and post-extubation time points in critically ill children.
• No extubation failure occurred in this study. Most respiratory and EIT parameters were similar in patients with abnormal compared to normal chest X-ray findings, with differences observed in TID and ODCL immediately after extubation.

**What are the implications of the main findings?**

• EIT can detect dynamic changes in lung volume and ventilation distribution around extubation in pediatric patients; however, its ability to identify extubation failure could not be evaluated in the absence of failed extubation events.
• The high rate of prophylactic HFNC/NIV use may influence extubation physiology and should be considered when interpreting EIT trends or designing future pediatric extubation studies.

**Abstract:**

**Background:** Extubation failure increases morbidity and mortality. Non-invasive ventilation (NIV), including high-flow nasal cannula (HFNC), can reduce reintubation rates. Current practice often involves prophylactic use of NIV post-extubation. Electrical Impedance Tomography (EIT) provides real-time monitoring of pulmonary distribution and ventilation. Recent adult studies suggest that EIT has potential in extubation failure prediction, but evidence in children is limited. Our objectives were to evaluate peri-extubation regional lung volume/distribution and to explore EIT-derived physiological changes and on post-extubation respiratory support patterns in critically ill children. **Methods:** A prospective observational study included intubated patients aged 1 month to 18 years in the PICU who were intubated for over 24 h. Vital signs and chest EIT were recorded pre-extubation (H0), immediately post-extubation (H1), at 30 min (H2), and at 4 h (H3). Patients were categorized by chest X-ray findings into abnormal or normal groups. **Results:** Among 209 ventilated patients, 54 were included. End-expiratory lung impedance (∆EELI), tidal impedance (TID), and the global inhomogeneity index (GI) demonstrated significant changes across predefined peri-extubation time points. Thirty-eight (70.4%) patients received HFNC or NIV immediately after extubation. No extubation failures occurred, precluding evaluation of extubation failure predictors. In the subgroup analyzed based on chest X-ray findings, differences in TID and ODCL were observed between patients with normal and abnormal chest X-rays immediately after extubation. **Conclusions:** The ∆EELI, TID, and GI demonstrated significant changes across predefined peri-extubation time points. In the absence of extubation failure events, the ability of EIT monitoring to evaluate extubation failure could not be assessed. The frequent use of prophylactic NIV support after extubation may have influenced post-extubation physiology.

## 1. Introduction

Extubation represents a critical transition in the management of critically ill children receiving invasive mechanical ventilation. Extubation failure is defined by the patient’s inability to sustain spontaneous breathing following extubation and needed reintubation within 48 h [1]. This condition is associated with unfavorable clinical outcomes, including prolonged ICU stay (LOS), extended mechanical ventilation (MV) duration, increased risk of tracheostomy [2], and increased morbidity and mortality [3,4]. The causes of extubation failure are typically multifactorial, involving age, neurological status [5], and duration of mechanical ventilation [6]. Successful extubation depends not only on these factors but also on dynamic changes in lung mechanics, gas exchange, and cardiopulmonary stability during the peri-extubation period.

Electrical Impedance Tomography (EIT) is a new-generation, non-invasive, real-time monitoring technique that measures the overall volume and regional distribution of pulmonary ventilation. This technology utilizes a series of 16 electrodes placed around the chest wall, creating a 2-dimensional cross-sectional image akin to a real-time computer tomography (CT) scan but with less radiation. EIT works by detecting differences in electrical impedance between tissue, gas, and liquid. It has been increasingly used to characterize ventilation distribution and to detect lung overdistention or collapse [7].

Several predictors for extubation failure have been identified through research and clinical experiences in the adult population, including the rapid shallow breathing index (RSBI), negative inspiratory force (NIF), blood gas analysis, and advanced monitoring techniques such as capnography, lung ultrasonography, and EIT. A recent study monitored forty intubated patients during spontaneous breathing trials (SBTs) at immediate, 2, and 6 h post-extubation using EIT. The results showed that the global inhomogeneity index (GI) and lung ultrasound score (LUS) were higher in the extubation failure group, while the regional ventilation delay (RVD) and the coefficient of variation (CoV) did not differ between the groups [8]. However, extrapolation of these findings to pediatric patients remains challenging. Children exhibit a distinct respiratory physiology compared to adults, characterized by smaller airway caliber, higher chest wall compliance, lower functional residual capacity, and increased susceptibility to airway collapse and atelectasis during weaning and after extubation. In addition, post-extubation respiratory support strategies such as high-flow nasal cannula (HFNC) or non-invasive ventilation (NIV) are frequently applied prophylactically in pediatric intensive care units and may influence lung volume and ventilation distribution differently from adults. Current EIT techniques have a limited ability to predict the rapid onset of atelectasis and lung collapse following weaning or extubation in children [9]. This study aimed to describe changes in regional lung volume and ventilation distribution measured by EIT before and after extubation and to explore EIT-derived physiological patterns associated with post-extubation respiratory support in critically ill children.

## 2. Materials and Methods

### 2.1. Study Design and Population

The study was a prospective cohort study conducted in the Pediatric Intensive Care Unit (PICU), Ramathibodi Hospital, Bangkok, Thailand, from February 2023 to January 2024. The PICU, which has 8 beds, accommodates both medical and surgical patients. Children aged 1 month to 18 years who had been intubated and received invasive mechanical ventilation for over 24 hand were ready for extubation by the attending physician were eligible for inclusion. Eligible patients underwent a spontaneous breathing trial for 1–2 h using pressure support ventilation. Pressure support was set at 10 cmH_2_O in children younger than 5 years and 8 cmH_2_O in those aged over 5 years, with PEEP maintained at 5–6 cmH_2_O and FiO_2_ 0.3–0.4. Extubation readiness was determined based on routine clinical assessment, including hemodynamic stability, adequate gas exchanges, appropriate ventilator settings, and appropriate neurological status; however, no standardized extubation protocol was applied. Patients were excluded if they had unintended extubation, open thoracotomies, or chest tube drainage, as these conditions preclude the use of EIT belts. The decision to initiate HFNC or NIV was determined by the attending physician and was not protocolized. Ethical approval for this study (COA.MURA2023/67) was obtained from the institution’s ethics committee, and written informed consent was obtained from the parents of all participating children. Patients were divided into two groups based on radiologists’ official chest X-ray findings: those with abnormal chest X-rays and those with normal chest X-rays.

### 2.2. Intervention and Data Collection

Electrical Impedance Tomography (EIT) was performed using the PulmoVista 500 (Dräger Medical, Lübeck, Germany), with patients in the supine position and breathing spontaneously. A proper-sized silicon 16-electrode belt was placed around the chest at the 4th to 5th intercostal spaces, with a reference electrode positioned on the abdomen. Each recording session lasted between 5 and 10 min. Vital signs and chest EIT parameters were assessed at several time points during the spontaneous breathing trials (SBTs) (H0) and after extubation, including immediately post-extubation (H1), at 30 min post-extubation (H2), and at 4 h post-extubation (H3).

We collected data on vital signs, including heart rate, respiratory rate, and oxygen saturation. The EIT data included tidal impedance (TID), the global inhomogeneity index (GI), the compliance change percentage variation (ODCL), change in end-expiratory lung impedance (ΔEELI), normalized deviation (NORMTID), surface of ventilated area (SURF), regional ventilation delay (RVD), and center of gravity (CG) of the right and left lungs at each time point.

### 2.3. Definitions

Various functional EIT parameters were described as follows [10,11,12]:

(1) Tidal impedance (TID) represents the distribution of ventilation for one averaged breath, calculated from the impedance change between the end and the beginning of inspiration.
(2) The global inhomogeneity index (GI) quantifies the homogeneity of ventilation distribution. It is calculated by summing the absolute differences between the median value of tidal variation and each pixel value, then divided by the sum of all impedance values.
(3) Change in end-expiratory lung impedance (ΔEELI) is defined as the difference in end-expiration lung impedance measured by EIT between two time points. ΔEELI have been showed to correlate linearly with changes in end-expiratory lung volume within the EIT sensitivity region [12].
(4) Compliance change percentage variation (ODCL) indicates changes in compliance at any positive end-expiratory pressure (PEEP), distinguishing pixels as overdistended or collapsed.
(5) Regional ventilation delay (RVD) represents the time lag between the global onset of inspiration and the point at which the regional impedance curve reaches a specified impedance change. It is quantified as a percentage by comparing the time to reach a certain impedance (volume) within different lung regions to the global rate.
(6) Normalized deviation from the maximal regional tidal variation (NORMTID) compares the tidal image of a given section with the maximal tidal variation map.
(7) Surface of ventilated area (SURF) describes the size and location of ventilated regions of the corresponding section.
(8) Center of gravity (CG), synonymous with the center of ventilation (COV), describes how ventilation is distributed between the ventral and dorsal lung regions. An average of two points divides each lung into two equal parts of the impedance variation along the anterior–posterior axis. A value of 50% corresponds to perfectly balanced ventilation.

### 2.4. Data and Statistical Analysis

EIT Image reconstruction using the software of the manufacturer (EIT Data Review Tool, Dräger Medical, Lübeck, Germany) was used. The EIT data were analyzed offline with customized software programmed with EIT Diag software version 1.6 from MATLAB R2015 (The MathWorks Inc., Natick, MA, USA).

Statistical analyses were conducted using IBM SPSS version 29.0.1 (IBM Corporation). Categorical variables were presented as numbers and percentages and analyzed using the Fisher exact or Chi-square tests. Continuous variables were expressed as medians (interquartile range). Two-tailed *t*-tests were employed for normally distributed continuous variables, while the Mann–Whitney U-test was utilized for non-parametric data. A significance level of *p* < 0.05 (two-sided) was deemed statistically significant for all tests.

### 2.5. Outcomes

The primary outcome was to describe the regional lung volume and distribution before and after extubation and to analyze differences across various time points. The secondary outcome was to explore EIT-derived physiological changes around extubation and on post-extubation respiratory support patterns in critically ill children.

## 3. Results

### 3.1. Populations

**Figure 1:**
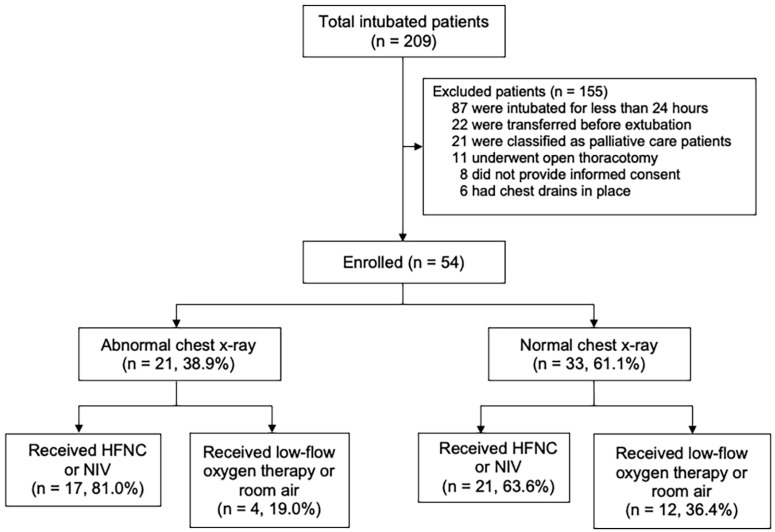
Study enrollment and patient classification. Flow diagram of patient enrollment, exclusions, and subgroup classification according to chest X-ray findings and post-extubation respiratory support. HFNC; high-flow nasal cannula; NIV; non-invasive ventilation.

A total of 155 patients, out of 209 intubated patients (74.2%), were excluded from the study (Figure 1). The most common reasons for exclusion were a duration of MV less than 24 h (n = 87), transfer to another facility before extubation (n = 22), palliative care status (n = 21), open thoracotomy (n = 11), chest tube drainage (n = 6), and lack of informed consent (n = 8).

The demographic data of all 54 included patients, including age, gender, severity of illness, ICU length of stay, indication for intubation, and pre-extubation blood gases and ventilator parameters, are summarized in Table 1.

### 3.2. Primary Outcome

**Figure 2:**
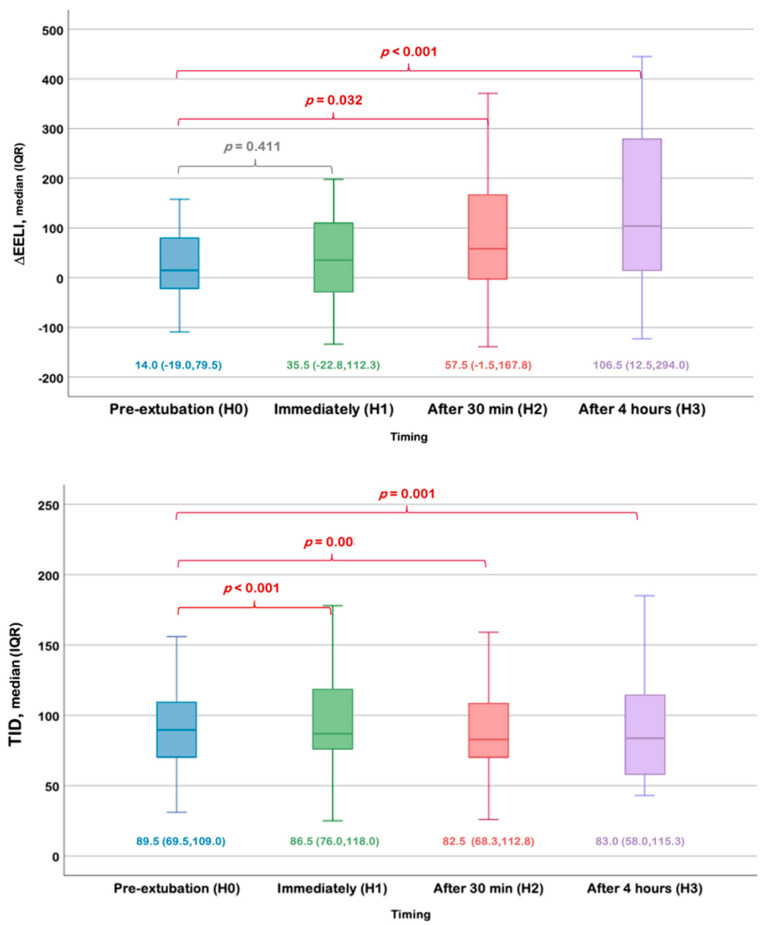
Comparison of the change in end-expiratory lung impedance (∆EELI) and tidal impedance (TID) at the predefined time points. Box-and-whisker plots showing ΔEELI (upper panel) and TID (lower panel) measured during the spontaneous breathing trial before extubation (H0), immediately after extubation (H1), at 30 min (H2), and at 4 h (H3). Data are presented as median and interquartile range. *p* values represent pairwise comparisons between time points.

The ΔEELI increased over time and became significantly higher than baseline, at 30 min and 4 h post-extubation (*p* < 0.05). The TID was significantly decreased over the time points compared to the values during SBTs (H0) (Figure 2). In the same direction, the GI increased from H0 to H3, respectively. All of these are summarized in Table 2.

Subgroup analysis (Figure 1 and Table 3) was performed among patients with positive chest X-ray findings, as reported by the radiologists, including atelectasis or consolidation. Twenty-one (38.9%) were managed by an individual physician with either HFNC or NIV, and four patients were receiving room air or low-flow oxygen therapy. The remaining 33 patients (61.1%) had normal chest X-ray findings. Twenty-one patients (63.6%) were managed with HFNC or NIV after extubation, while twelve patients were receiving room air or low-flow oxygen therapy. In the subgroup analysis, differences in blood gas values were observed before extubation. In patients with abnormal chest X-ray findings, the median (interquartile range, IQR) pCO_2_ was 41.80 (38.05–47.35) compared to 37.60 (34.50–41.85) mmHg in those with normal chest X-rays (*p* = 0.009). Similarly, the median (IQR) ratio of arterial oxygen partial pressure to fractional inspired oxygen (PF ratio) was lower in the abnormal chest X-ray group at 280.00 (137.09–390.42) than in the normal chest X-ray group at 476.67 (256.25-532.92) (*p* = 0.017). However, baseline characteristics, other blood gases, or ventilator parameters did not differ between the groups. Thirty-eight patients (70.4%) received NIV or HFNC immediately after extubation, with no experience of extubation failure. Comparison between the abnormal and normal chest X-ray groups revealed no significant difference in respiratory and EIT parameters, except for the TID and ODCL, which differed significantly between groups immediately after extubation (*p* = 0.029, 0.024, respectively) (Table 4).

### 3.3. Secondary Outcomes

Based on a previous adult study reporting an ODCL value greater than 21 as a marker of reduced lung compliance [13], this cutoff was applied in the present study solely as exploratory reference to describe the post-extubation support pattern. Of the patients who had abnormal chest X-ray findings (n = 21), 17 patients received prophylactic NIV or HFNC after extubation. Among these patients, 12 (57.1%) had ODCL values greater than 21, whereas 5 (23.8%) had ODCL < 21 and still received prophylactic HFNC/NIV. The remaining four patients received room air or low-flow oxygen therapy.

## 4. Discussion

This study represents the first utilization of EIT during SBTs and post-extubation in pediatric patients. We observed significant peri-extubation changes in ΔEELI, which likely reflect dynamic changes in end-expiratory lung volume (EELV) within the EIT sensitivity region over the time. These findings align with previous studies in adults. However, although ΔEELI closely correlates with changes in EELV, as demonstrated in previous literature, we were unable to confirm this correlation in our study due to limitations of the ventilator machine that we used. The observed reduction in TID immediately after extubation likely reflects the expected shift from controlled ventilation with relatively higher tidal volumes to spontaneous breathing with lower tidal volumes. Tidal impedance decreased from baseline at time points corresponding with an increase in the global inhomogeneity index. A rise in GI indicates a shift towards greater lung inhomogeneity, which was associated with a decrease in tidal impedance. This phenomenon may suggest the presence of collapse or atelectasis in the dependent lung regions, particularly evident immediately after extubation, as mentioned in previous studies of preterm infants [14]. This was observed through regional ventilation delay (RVD) and the significant decrease in normalized deviation from maximal regional tidal variation (NORMTID) (*p* < 0.001) at the same time. Our study showed the progressive rise in ΔEELI over the first 4 h may represent gradual improvement in lung aeration, possibly facilitated by prophylactic HFNC/NIV use after extubation. However, other EIT and respiratory parameters did not exhibit significant differences at these time points.

A prior adult study reported that an ODCL greater than 21 during 15-min SBTs was significantly associated with extubation failure in adults [15]. In the absence of pediatric-specific validation and extubation failure events in our cohort, this cutoff was used solely as an exploratory physiological marker to describe post-extubation respiratory support pattern rather than as a predictive threshold. Our results showed that patients with higher ODCL values more frequently received NIV or HFNC, while several patients with lower ODCL values also received prophylactic respiratory support. The routine use of early NIV or HFNC in our center may have prevented clinical deterioration but also limited the ability to evaluate EIT-derived predictors of extubation failure. Further studies with more selective use of post-extubation support or stratified protocols guided by EIT parameters may help determine whether ODCL or related EIT metrics can identify children who truly benefit from early NIV or HFNC versus those who can safely avoid unnecessary interventions.

This study has several limitations. First, the relatively small sample size with single center may reduce the statistical power and limit the generalizability of our finding. Second, extubation readiness and post-extubation respiratory support were determined by physician judgment without a standardized extubation protocol, which may have introduced variability in patient selection and management decisions. Third, no extubation failures occurred in our cohort, which prevented the evaluation of EIT-derived predictors and limited the conclusions that could be drawn regarding extubation outcomes. Fourth, multiple comparisons were performed across predefined time points without formal adjustment; therefore, the findings should be interpreted as exploratory rather than confirmatory. Finally, although EIT is generally non–operator dependent, potential bias related to belt displacement remains a concern. To minimize this, we carefully maintained belt position throughout the study by marking the skin and loosening, rather than removing, the belt when necessary.

## 5. Conclusions

This study is the first study to apply EIT for the monitoring of both SBTs and the post-extubation period in children. Significant changes in ΔEELI, tidal impedance, and the global inhomogeneity index were observed across time points, demonstrating dynamic alterations in lung volume and ventilation distribution around extubation. Further studies with a more selective use of non-invasive support are needed to clarify the predictive value of EIT in pediatric extubation.

## Figures and Tables

**Table 1:** Baseline characteristics of the study population.

Variables	All Patients (n = 54) [Median (IQR)]
Sex: male *	28 (51.9%)
Age (months)	29.0 (12.0, 78.5)
BSA (m^2^)	0.55 (0.37, 0.89)
PRISM III	1.5 (0, 7.5)
Mechanical ventilator (days)	3.0 (1.8, 5.0)
Length of ICU stay (days)	5.0 (3.0, 8.0)
Immune status *	
• Immunocompetent host	47 (87.0)
• Immunocompromised host	7 (13.0)
Reason for ICU admission *	
• Respiratory causes	24 (44.5)
• Post operation	9 (16.7)
• Neurological causes	7 (13.0)
• Cardiac causes	7 (13.0)
• Infectious causes	4 (7.1)
• Hematologic causes	3 (5.6)
Indication for intubation *	
• Post operation	23 (42.5)
• Respiratory causes	13 (24.1)
• Neurological causes	8 (14.8)
• Cardiac causes	7 (13.0)
• Other causes	3 (5.5)
Arterial blood gas before extubation	
• pH	7.42 (7.38, 7.46)
• pCO_2_, mmHg	40.90 (35.70, 42.30)
• PF ratio	382.50 (219.90, 520.00)
Ventilator parameters	
• Tidal volume per bodyweight (ml/kg)	10.00 (8.20, 13.30)
• P0.1	0.70 (1.30, 2.00)
• Dynamic compliance (Cdyn)	15.45 (8.88, 19.85)

* Number (percentage, %). BSA, body surface area; PRISM III, Pediatric Risk of Mortality III; ICU, intensive care unit; pCO_2_, the partial pressure of carbon dioxide; PF ratio, the ratio of arterial oxygen partial pressure to fractional inspired oxygen; P0.1, negative pressure at airway opening 100 msec after the initiation of an inspiratory effort.

**Table 2:** Respiratory and Electrical Impedance Tomography parameters during spontaneous breathing trial and at predefined time points after extubation.

Parameters [Median (IQR)]	During Spontaneous Breathing Trial (SBT) (H0)	After Extubation (Compared to Baseline SBT)		
		Immediate (H1)	30 min (H2)	4 h (H3)
Respiratory parameters				
Heart rate (beat per minute)	110 (98, 120)	110 (95, 130)	105 (90, 123)	105 (95, 118)
Respiratory rate (per minute)	24 (18, 30)	26 (22, 32)	26 (24, 30)	26 (22, 28)
Oxygen saturation (%)	99 (99, 100)	99 (98, 100)	99 (98, 100)	99 (99, 100)
Electrical Impedance Tomography (EIT) parameters				
TID	89.5 (69.5, 109.0)	86.5 (76.0, 118.0)	82.5 (68.3, 112.8)	83.0 (58.0, 115.3)
	*p* value	<0.001	0.003	0.001
GI	48.0 (43.5, 60.0)	49.0 (45.0, 59.3)	49.0 (44.0, 60.3)	54.0 (46.8, 68.5)
	*p* value	<0.001	<0.001	<0.001
∆EELI	14.0 (−19.0, 79.5)	35.5 (−22.8, 112.3)	57.5 (−1.5, 167.8)	106.5 (12.5, 294.0)
	*p* value	0.411	0.032	<0.001
RVD	8.5 (6.2, 10.2)	7.9 (6.4, 9.8)	8.3 (5.9, 10.4)	8.6 (6.3, 10.4)
	*p* value	<0.001	<0.001	0.194
ODCL	30.5 (24.75, 42.0)	27 (14.8, 40.3)	31.5 (20.8, 40.8)	32.0 (22.0, 48.0)
	*p* value	0.103	0.122	0.358
NORMTID	34.0 (23.8, 48.5)	32.0 (25.0, 52.8)	36.0 (26.0, 53.0)	47.0 (29.8, 68.3)
	*p* value	<0.001	<0.001	<0.001
SURF	444.0 (404.5, 493.0)	451.0 (401.5, 496.8)	443.0 (378.0, 510.3)	426.0 (378.5, 471.8)
	*p* value	<0.001	<0.001	<0.001
CG_Right	51.5 (47.8, 56.0)	51.0 (47.0, 54.3)	50.5 (47.0, 54.0)	50.0 (47.0, 54.0)
	*p* value	<0.001	<0.001	<0.001
CG_Left	52.0 (49.0, 55.3)	51.0 (47.0, 53.3)	52.0 (47.0, 54.3)	50.0 (46.0, 54.3)
	*p* value	<0.001	<0.001	0.100

TID, tidal impedance; GI, global inhomogeneity index; ΔEELI, change in end-expiratory lung impedance; RVD, regional ventilation delay; ODCL, compliance change percentage variation; NORMTID, normalized deviation from the maximal regional tidal variation; SURF, surface of ventilated area; CG, center of gravity.

**Table 3:** Baseline characteristics of the study population according to chest X-ray findings (abnormal CXR versus normal CXR).

Variables Median (IQR)	Abnormal CXR * [n = 21 (38.9)]	Normal CXR * [n = 33 (61.1)]	*p* Value
Sex: male *	11 (52.4)	17 (51.5)	0.951
Age (months)	31.0 (14.0, 70.0)	23.0 (10.5, 98.0)	0.435
BSA (m^2^)	0.62 (0.37, 0.81)	0.49 (0.37, 0.98)	0.683
PRISM III	0 (0, 2.5)	2.0 (0, 8.5)	0.049
Mechanical ventilator (days)	3.0 (2.0, 6.5)	3.0 (1.0, 5.0)	0.175
Length of ICU stay (days)	5.0 (4.0, 7.5)	5.0 (2.5, 8.5)	0.655
Immune status *			
• Immunocompetent host	20 (95.2)	27 (81.8)	0.156
• Immunocompromised host	1 (4.8)	6 (18.2)	
Reason for ICU admission *			
• Post-operation	0	9 (27.3)	0.009
• Respiratory causes	20 (95.2)	4 (12.1)	
• Neurological causes	0	7 (21.2)	
• Cardiac causes	1 (4.8)	6 (18.2)	
• Hematologic causes	0	3 (9.1)	
• Infectious causes	0	3 (9.1)	
Indication for intubation *			
• Post operation	0	13 (39.4)	0.315
• Respiratory causes	19 (90.4)	4 (12.1)	
• Neurological causes	1 (4.8)	7 (21.2)	
• Cardiac causes	1 (4.8)	6 (18.2)	
• Infectious causes	0	3 (9.1)	
Blood gas before extubation			
• pH	7.42 (7.37, 7.45)	7.55 (7.39, 7.48)	0.084
• pCO_2_, mmHg	41.80 (38.05, 47.35)	37.60 (34.50, 41.85)	0.009
• PF ratio	280.00 (137.09, 390.42)	476.67 (256.25, 532.92)	0.017
Ventilator parameters			
• Tidal volume per bodyweight (mL/kg)	9.90 (8.10, 12.55)	10.30 (8.25, 13.95)	0.783
• Occlusion pressure (P0.1)	1.30 (0.60, 1.85)	1.30 (0.75, 2.00)	0.756
• Dynamic compliance (Cdyn)	16.00 (7.95, 21.90)	15.40 (8.45, 19.50)	0.979

* Number (percentage, %). PRISM III, Pediatric Risk of Mortality III; ICU, intensive care unit; pCO_2_, the partial pressure of carbon dioxide; PF ratio, the ratio of arterial oxygen partial pressure to fractional inspired oxygen; P0.1, negative pressure at airway opening 100 msec after the initiation of an inspiratory effort.

**Table 4:** Respiratory and Electrical Impedance Tomography parameters during spontaneous breathing trial and after extubation stratified by chest X-ray findings (abnormal CXR versus normal CXR).

Parameters	Subgroup	During Spontaneous Breathing Trials (SBT) (H0)	After Extubation		
			Immediate (H1)	30 min (H2)	4 h (H3)
Respiratory parameters					
Heart rate (beat per minute)	Abnormal CXR	110 (91, 123)	110 (95, 130)	106 (94, 132)	110 (92, 120)
	Normal CXR	110 (100, 116)	110 (95, 126)	105 (85, 120)	105 (95, 113)
	*p* value		0.516	0.163	0.262
Respiratory rate (per minute)	Abnormal CXR	22 (20, 26)	26 (23, 35)	26 (21, 31)	26 (22, 30)
	Normal CXR	24 (18, 30)	26 (22, 31)	26 (24, 30)	24 (22, 28)
	*p* value		0.549	0.630	0.768
Oxygen saturation (%)	Abnormal CXR	100 (99, 100)	100 (99, 100)	100 (99, 100)	100 (100, 100)
	Normal CXR	100 (100, 100)	100 (100, 100)	100 (100, 100)	100 (100, 100)
	*p* value		0.878	0.546	0.653
EIT parameters					
TID	Abnormal CXR	93.0 (80.0, 109.0)	82.0 (71.5, 114.0)	84.0 (66.0, 134.0)	96.0 (63.0, 144.5)
	Normal CXR	84.0 (68.0, 108.0)	83.0 (88.0, 126.5)	82.0 (67.0, 102.5)	73.0 (58.0, 112.5)
	*p* value		0.029	0.873	0.114
GI	Abnormal CXR	53.0 (46.0, 72.0)	59.0 (47.0, 81.5)	58.0 (42.0, 78.5)	58.0 (45.5, 73.5)
	Normal CXR	45.0 (42.0, 52.5)	46.0 (45.0, 53.0)	47.0 (44.0, 52.0)	51.0 (47.0, 59.5)
	*p* value		0.378	0.936	0.095
∆EELI	Abnormal CXR	30.0 (−28.0, 154.0)	94.0 (−31.0, 156.0)	97.0 (2.5, 221.5)	127.0 (−16.5, 308.0)
	Normal CXR	13.0 (−18.0, 224.5)	31.0 (−24.5, 99.5)	34.0 (−26.5, 169.5)	90.0 (18.5, 307.5)
	*p* value		0.148	0.929	0.965
∆EELV	Abnormal CXR	54.0 (−48.5, 245.9)	74.2 (−36.2, 299.1)	106.2 (−0.7, 398.1)	188.0 (−31.8, 704.5)
	Normal CXR	14.0 (−44.1, 29.5)	31.5 (−29.7, 102.5)	−1.9 (−129.0, 78.2)	89.6 (16.0, 332.1)
	*p* value		0.090	0.409	0.894
RVD	Abnormal CXR	7.6 (6.0, 10.6)	8.0 (5.3, 10.2)	8.8 (7.2, 10.6)	8.4 (5.7, 11.2)
	Normal CXR	8.7 (6.8, 10.2)	7.7 (6.4, 9.9)	7.7 (5.4, 9.9)	8.7 (6.5, 10.3)
	*p* value		0.338	0.114	0.845
ODCL	Abnormal CXR	30.0 (22.5, 38.5)	34.0 (17.5, 50.5)	32.0 (19.5, 50.5)	28.0 (18.0, 46.0)
	Normal CXR	31.0 (25.0, 43.0)	21.0 (13.5, 34.0)	29.0 (21.5, 37.5)	34.0 (25.0, 48.0)
	*p* value		0.024	0.558	0.172
NORMTID	Abnormal CXR	40.0 (28.0, 77.0)	45.0 (24.5, 83.0)	50.0 (24.5, 80.5)	51.0 (29.5, 73.0)
	Normal CXR	30.0 (23.0, 40.0)	31.0 (25.5, 42.0)	35.0 (27.0, 43.5)	46.0 (32.0, 53.5)
	*p* value		0.722	0.316	0.056
SURF	Abnormal CXR	434.0 (352.0, 489.5)	440.0 (324.0, 482.5)	419.0 (334.5, 494.0)	412.0 (360.0, 479.5)
	Normal CXR	457.0 (418.5, 498.5)	455.0 (418.5, 507.0)	456.0 (404.0, 513.5)	431.0 (380.0, 474.5)
	*p* value		0.922	0.929	0.051
CG_Right	Abnormal CXR	51.0 (45.0, 54.5)	49.0 (41.5, 54.0)	50.0 (47.5, 53.0)	50.0 (46.0, 53.5)
	Normal CXR	54.0 (48.0, 56.5)	52.0 (47.5, 55.0)	51.0 (47.0, 54.0)	49.0 (47.0, 54.5)
	*p* value		0.048	0.859	0.593
CG_Left	Abnormal CXR	53.0 (48.5, 57.0)	52.0 (49.5, 54.0)	52.0 (48.0, 54.5)	51.0 (46.5, 54.5)
	Normal CXR	51.0 (49.0, 55.0)	51.0 (46.5, 53.0)	52.0 (46.5, 54.5)	49.0 (46.0, 53.5)
	*p* value		0.456	0.291	0.708

CXR, chest X-ray; TID, tidal impedance; GI, global inhomogeneity index; ΔEELI, change in end-expiratory lung impedance; RVD, regional ventilation delay; ODCL, compliance change percentage variation; NORMTID, normalized deviation from the maximal regional tidal variation; SURF, surface of ventilated area; CG, center of gravity.

## Data Availability

The data that support the findings of this study are available on request from the corresponding author. The data are not publicly available due to privacy or ethical restrictions.

## Abbreviations

The following abbreviations are used in this manuscript:

AAP	American Academy of Pediatrics
ABG	Arterial blood gas
BSA	Body surface area
CG	Center of gravity
CoV	Coefficient of variation
CT	Computer tomography
ΔEELI	Delta end-expiratory lung impedance
ΔEELV	End-expiratory lung volume
EIT	Electrical Impedance Tomography
FRC	Functional residual capacity
GI	Global inhomogeneity index
HFNC	High-flow nasal cannula
ICU	Intensive care unit
LOS	Length of stay
LUS	Lung ultrasound score
MV	Mechanical ventilation
NIF	Negative inspiratory force
NIV	Non-invasive ventilation
NORMTID	Normalized deviation from the maximal regional tidal variation
ODCL	Compliance change percentage variation
pCO_2_	Partial pressure of carbon dioxide
PF ratio	The ratio of arterial oxygen partial pressure to fractional inspired oxygen
PEEP	Positive end-expiratory pressure
PRISM III	Pediatric Risk of Mortality III score
RSBI	Rapid shallow breathing index
RVD	Regional ventilation delay
SBT	Spontaneous breathing trials
SURF	Surface of ventilated area
TID	Tidal impedance
